# Chili pepper extracts, capsaicin, and dihydrocapsaicin as potential anticancer agents targeting topoisomerases

**DOI:** 10.1186/s12906-024-04394-5

**Published:** 2024-02-21

**Authors:** Terézia Hudáková, Martina Šemeláková, Peter Očenáš, Mária Kožurková, Kristína Krochtová, Simona Sovová, Zuzana Tóthová, Zuzana Guľášová, Peter Popelka, Peter Solár

**Affiliations:** 1 Department of Medical Biology, Faculty of Medicine, Pavol Jozef Šafárik University in Košice, Trieda SNP 1, 040 11 Košice, Slovakia; 2 Department of Chemistry, Biochemistry and Biophysics, University of Veterinary Medicine and Pharmacy in Košice, Komenského 73, 041 81 Košice, Slovakia; 3 Department of Biochemistry, Faculty of Science, Pavol Jozef Šafárik University in Košice, Moyzesova 11, 040 01 Košice, Slovakia; 4 Center of Clinical and Preclinical Research MEDIPARK, Faculty of Medicine, Pavol Jozef Šafárik University in Košice, Trieda SNP 1, 040 11 Košice, Slovakia; 5 Department of Food Hygiene, Technology and Safety, University of Veterinary Medicine and Pharmacy in Košice, Komenského 73, 041 81 Košice, Slovakia

**Keywords:** Chili pepper extract, Capsaicin, Dihydrocapsaicin, Topoisomerases inhibition

## Abstract

DNA topoisomerases regulate conformational changes in DNA topology during normal cell growth, such as replication, transcription, recombination, and repair, and may be targeted for anticancer drugs. A DNA topology assay was used to investigate DNA-damaging/protective activities of extracts from Habanero Red (HR), Habanero Maya Red (HMR), Trinidad Moruga Scorpion (TMS), Jalapeno (J), Serrano pepper (SP), Habanero Red Savina (HRS), Bhut Jolokia (BJ), and Jamaica Rosso (JR) peppers, demonstrating their inhibitory effect on the relaxation of pBR by Topo I. DNA topoisomerase II (Topo II) is proven therapeutic target of anticancer drugs. Complete inhibition of Topo II was observed for samples TMS, HR, and HMR. Extracts J and SP had the lowest capsaicin and dihydrocapsaicin content compared to other peppers. HR, HMR, TMS, J, S, HRS, BJ, JR extracts showed the anticancer effect, examined by MTS and xCell assay on the in vitro culture of human colon carcinoma cell line HCT116.

**Supplementary Information:**

The online version contains supplementary material available at 10.1186/s12906-024-04394-5.

## Introduction

Cancer disease is a serious health and social problem. Despite therapeutic advances, cancer is the second leading cause of morbidity and mortality worldwide (https://www.who.int/health-topics/cancer#tab=tab_1). Although treating cancer with chemotherapy and radiotherapy is effective, it is associated with serious side effects, such as drug resistance or non-selectivity [1]. These problems illustrate the need to develop new, more effective anticancer therapies and safer agents [2]. Natural products or their direct derivatives play an important role in the discovery of new drugs for the treatment of cancer [3]. The plant compounds have different inhibitory effects on cancer onset, development, progression, and metastasis [4, 5]. Plants of the genus *Capsicum*, belonging to the family *Solanaceae*, are an important source of biologically active substances [6]. We currently recognize 25 wild species and five domesticated species in the genus *Capsicum* [7], *Capsicum annuum*, *Capsicum frutescens*, *Capsicum chinense*, *Capsicum baccatum*, and *Capsicum pubescens* [8]. In addition to their use in gastronomy, peppers are also excellent producers of secondary metabolites, which have various pharmacological properties and contain cytotoxic compounds [9].

A characteristic feature of wide varieties of peppers is their intense pungency caused by a group of bioactive phytochemicals, capsaicinoids, classified as alkaloids. They are vanilylamides derived from branched-chain C8-C11 (E) -monocline fatty acids and branched-chain or straight-chain saturated fatty acids [10]. One of the capsaicinoids, capsaicin (48.6%) (CAP) is the most abundant compound in chili peppers, followed by 6,7-dihydrocapsaicin (36%) (DHK), nordihydrocapsaicin (7.4%), homodihydrocapsaicin (2%), and homocapsaicin (2%). CAP (trans-8-methyl-N-vanillyl-6-non enamide) is a crystalline, lipophilic, colorless, and odorless alkaloid soluble in fats, alcohols, and oils [11, 12]. Many studies have shown that capsaicinoids have a wide range of biological and physiological effects. Capsaicinoid biosynthesis and accumulation is a genetically determined trait in chili pepper fruits as different cultivars or genotypes, where gene expression has identified candidate genes possibly involved in capsaicinoid biosynthesis [10, 13]. CAP has analgesic [14, 15] and anti-inflammatory effects [16], decreases the prevalence of obesity [17] and metabolic syndrome, improves gastrointestinal [18] and cardiovascular symptoms [19], and is characterized by antitumor activity [20, 21]. The nutritional, and anti-obesity properties of different chili peppers was presented by Azlan et al. [22]. Due to ability of CAP to mediate cell cycle arrest and induce cell apoptosis in in vitro experiments, it reduced the growth of human leukemia cells [23], skin tumor cells [24], prostate [25, 26], bladder [27], stomach [28, 29], colon [30], nasopharynx [31], liver [32], lung [33], and breast cancer [34]. Capsaicin can modify the function of many genes associated with the lifespan of cancer cells, initiating apoptosis, arresting cell growth, and suppressing angiogenesis and metastasis [35, 36]. By inducing apoptosis in cancer cell lines, healthy cells remain intact [37]. Several studies have shown that new combination therapies with various phytochemicals and chemopreventive drugs can induce increased antitumor activity through an additive or synergistic effect [38]. Capsaicinoids potentiate the chemotherapeutic effect and relieve pain in cancer patients. CAP acts synergistically with other anticancer agents; thus, it can be used with other chemotherapeutic agents in cancer treatment [39–41]. Colorectal cancer is one of the most commonly diagnosed diseases in the world [42]. The incidence of this disease is closely related to the composition of the diet and the amount of vegetables consumed. Currently, preclinical studies testing the anticancer effects of CAP on colon cancer are lacking [43–45].

DNA topoisomerases regulate conformational changes in DNA topology during normal cell growth, such as DNA replication, transcription, recombination, and DNA repair [46]. They are also targets for several anticancer drugs [47, 48]. Topoisomerase inhibitors interfere with human topoisomerases, or they can act as inhibitors without tumor cell toxicity [49].

The aim of our work was to investigate the DNA-damaging/protective activities of the studied chili extracts. The DNA topology was studied with electrophoretic detection of topological changes induced in plasmid DNA. We hypothesized that an extract of CAP, DHK, and other varieties of chili peppers would influence both Topo I and II and exploit the ability to act on two distinct enzymatic targets, thereby maximizing the potential therapeutic effects. The biological activity of extracts was assessed using an MTT assay of the human colon cancer cell line HCT-116, potentially usable in cancer therapy and drug screening.

## Materials and methods

### Sample processing

All analyzed types of chili peppers: Habanero Red (HR), Habanero Maya Red (HMR), Trinidad Moruga Scorpion (TMS), Jalapeno (J), Serrano pepper (SP), Habanero Red Savina (HRS), Bhut Jolokia (BJ), Jamaica Rosso (JR) were grown and harvested at the Department of Food Hygiene, Technology and Safety of the University of Veterinary Medicine and Pharmacy in Košice. The cultivated peppers were dried in a laboratory oven with ventilation at 40 ± 5 °C. Before drying, the chili peppers were cut in half or quarters (depending on the size) in order to speed up the drying and to avoid undesired changes. The peppers were dried together with the placenta and seeds. After drying, they were stored in a closed glass container in a dry, dark place until analysis.

### Extraction of capsaicinoids

Completely dried fruits of various varieties of peppers were ground completely on an electric stainless steel mixer. From each pepper sample 0.2 g of ground pepper was weighed into 10 mL volumetric flasks and 2 ml of 96 (v/v) ethanol were added. The mixture was mixed on a Vortex homogenizer and placed in an ultrasonic bath for 5 minutes. After homogenization, the samples were macerated for 24 hrs in a dry and dark place under laboratory conditions. After the indicated time, the individual samples were filtered through filter paper into 10 mL volumetric flasks, washed with absolute ethanol and made up to 10 mL with the same solvent. The extracts were stored in sealed flasks at 5 °C in a refrigerator. Samples were filtered through a membrane syringe filter (Q-Max® RR Siringe Filters, Frisenette, 25 mm, 0.22 μm PVDF) prior to HPLC analysis. If CAP or DHK concentrations were outside the calibration range, the samples were diluted with absolute ethanol.

### HPLC analysis

CAP and DHK standards were purchased from Sigma-Aldrich (USA), absolute ethanol from Emparta (Germany) and HPLC grade acetonitrile from Fisher (UK). The concentration of CAP and DHK in the extracts was determined using a Dionex UltiMate 3000 RS with a diode array detector (DAD) and a programmable Chromeleon Chromatography Data System, version 7.2 (Thermo Fisher Scientific, Germany). HPLC analysis was performed using a Polaris 5 column (C18-A 250 × 4.6 mm, 5 m, under isocratic conditions, at 40 °C and flow rate 1 mL.min^−1^. The sample was dosed using an autosampler and its volume was 10 L. The mixture of acetonitrile and water (70:30, v/v) was used as the mobile phase. CAP and DHK were measured with UV detector (DAD) at 282 nm. The quantification and HPLC method validation was based on the calibration curve fitting by linear regression analysis. Linear correlation between the peak area and the applied concentration was found in the concentration range 5–500 μg.mL^−1^, as confirmed by the correlation coefficient (0.99902 for CAP and 0.99932 for DHK). The x-axis in the graphical dependence represented the concentration of CAP or DHK and the y-axis was the peak area in the chromatographic record. The mean values for the regression equation were y = 0.027.x + 0.2049 for CAP and y = 0.0067x + 0.0057 for DHK.

### Nuclease activity

A nuclease activity study was performed prior to the experiments, which confirmed that none of the samples were able to cleave plasmid DNA and that the ethanol content did not affect the plasmid. Nuclease activity of selected molecules were studied using isolated plasmid pUC19 (isolated by the alkaline lysis method in our laboratory). Mixture of pUC19 in TE buffer (10 mM Tris, 1 mM EDTA, pH 8.0, 2 μl) (Sigma-Aldrich), 10 mM Tris-HCl buffer (pH 7.4, 25 μL) (Sigma-Aldrich) and studied compounds (3 μL) in final concentration 1/10 of stock solution were incubated at 37 °C for 18 hrs. After incubation solution of bromophenol blue and xylene violet (3 μL) and samples were subjected on 1.0% v/v agarose (Sigma-Aldrich) gel. Electrophoresis ran 4 h at 35 V in 1xTAE (40 mM Tris, 20 mM acetic acid glacial (Centralchem), 1 mM EDTA) (Sigma-Aldrich) then it was stained with ethidium bromide for 15 min and destained in deionized water for 7 min. Electrophoretic record was photographed with electrophoretic system SYNGEN and processed with GeneSnap program.

### Decatenation assay for topoisomerase II

Topoisomerase II (Topo II) decatenation assay was carried out according to the Inspiralis protocol using kinetoplast DNA (kDNA, 200 ng) in TE buffer (10 mM Tris-HCl (pH 8.0), 1 mM EDTA) (Sigma-Aldrich) and appropriate amount of diluted human topoisomerase IIa (hTop IIa, 1.5 U) enzyme in dilution buffer (50 mM Tris-HCl (pH 7.5), 100 mM NaCl, 1 mM DTT, 0.5 mM EDTA, 50% v/v glycerol, 50 μg/mL albumin (Inspiralis). Assay was conducted in final concentration 1/10 of stock solutions (3 μL from stock solutions, in the case of CAP we used two stock solutions – 0.5 mg/mL and 1.0 mg/mL). Samples were prepared using a mixture of appropriate assay buffer (50 mM Tris-HCl (pH 7.5), 125 mM NaCl, 10 mM MgCl_2_, 5 mM DTT, 100 μg/mL albumin, supplied as 10 × (Inspiralis)), 30 mM ATP (final concentration 1 mM) and deionized water in final volume 30 μL. Samples were incubated 30 min at 37 °C and after that reaction was stopped with STEB (30 μL, 40% v/v sucrose (Centralchem), 100 mM Tris-HCl (pH 8.0), 10 mM EDTA, 0.5 g/dm^3^ bromophenol blue (Sigma-Aldrich)) and purified with chloroform:isoamylalkohol (Centralchem) (30 μL, 24:1) solution and subjected to the 1% v/v agarose gel in 1× TAE (40 mM Tris, 20 mM acetic acid glacial (Centralchem), 1 mM EDTA (Sigma-Aldrich)). Dilution of ethanol (% v/v) in samples for Topo I a Topo II was 3 μl ethanol/ 30 μl sample. Electrophoresis ran 4 hrs at 35 V and then agarose gel was stained with ethidium bromide solution and destained in water. Electrophoretic record was documented using UV light.

### Relaxation assay for topoisomerase I

Impact of molecules on relaxation ability of topoisomerase I was studied on human topoisomerase I (hTopo I, Inspiralis) on plasmid pBR322 (Inspiralis). Mixture of plasmid (0.5 μg) in TE (10 mM Tris-HCl (pH 7.5), 1 mM EDTA), diluted hTopoI (0.5 U) in dilution buffer (10 mM Tris-HCl (pH 7.5), 1 mM DTT, 1 mM EDTA, 50% v/v glycerol, 50 μg/mL albumin (Inspiralis)) and studied compounds in final concentrations of 1/10 of stock solutions (3 μL from stock solution, in case of CAP were used two stock solutions – 0.5 mg/mL and 1.0 mg/mL) were incubated at 37 °C for 30 minutes in 1 × concentrated assay buffer (20 mM Tris-HCl (pH 7.5), 200 mM NaCl, 0.25 mM EDTA, 5% glycerol, 50 μg/mL albumin, supplied as 10× stock (Inspiralis)) and deionized water in the final volume of 30 uL. After incubation reaction was stopped with STEB (30 μL, 40% v/v sucrose (Centralchem), 100 mM Tris-HCl (pH 8.0), 10 mM EDTA, 0.5 g/dm3 bromophenol blue (Sigma-Aldrich)) and samples were purified with chloroform:isoamyl alcohol (Centralchem) (30 μL, 24:1) and upper layer of samples was subsequently subjected on 1% v/v agarose gel. Electrophoresis ran for 15 min at 20 V to allow subjected samples to penetrate the gel and then continued for 4 hrs at 35 V in 1 × TAE buffer (40 mM Tris, 20 mM acetic acid glacial (Centralchem), 1 mM EDTA (Sigma-Aldrich)). Then agarose gel was stained with ethidium bromide solution (15 min) and destained with deionized water (10 min). Electrophoretic record was visualized by UV light, photographed by SYNGEN system and processed in GeneSnap program.

### Cell line

Human colon carcinoma cell line HCT116 (ATCC® CCL-247™) was cultured in RPMI medium supplemented by antibiotics (100 U/ml penicillin + 100 μg/mL penicilin-streptomycin) and 10% of FBS (fetal bovine serum) in the presence of 5% CO_2_ in a humidified atmosphere at 37 °C. If 5 × 10^6^ cells were plated onto a 75 cm^2^ flask, the culture reaches 70-90% confluency in 2-3 days and was ready to split or harvest for experiments. To determine the linear range of each assay, six cell densities ranging from 50 to 10 000 cells/well were plated into sterile 96-well plates and incubated for 24, 48 or 72 hrs.

### MTS assay

MTS assay (3-(4,5-dimethylthiazol-2-yl)-5-(3-carboxymethoxyphenyl)-2-(4-sulfophenyl) -2H-tetrazolium) as indicators of metabolically active mitochondria overestimated the number of viable cells. MTS was used for determining the number of viable cells in proliferation, cytotoxicity, or chemosensitivity. HCT116 cells were seeded at 5000 cells per well into 96-well microplates, after 24 hrs incubation treated with extracts of individual samples (extracts of peppers), CAP or DHK. After 24 hrs incubation of HCT116 cells, medium was removed and replaced with RPMI containing 10% fetal bovine serum (FBS) and extracts of dry extracts in ethanol peppers: HR, HMR, TMS, J, S, HRS, BJ, JR in dilution 100×, 500×,1000× and incubated at 37 °C and 5% CO_2_ for 24 hrs. Control group (cells HCT116) was not affected by extracts, CAP1 - CAP6 concentrations of CAP at 10 μM, 25 μM, 50 μM, 100 μM, 150 μM, 200 μM. DHK1 - DHK6 concentrations of DHK at 10 μM, 25 μM, 50 μM, 100 μM, 150 μM, 200 μM. The MTS assay was performed at 48 hrs and 72 hrs. After the 24 hrs exposure to the cells, 25 μL of CellTiter 96, AQueous One Solution Cell Proliferation Assay (MTS) (Promega, Madison, WI, USA) was added to the cell culture medium, and incubated for 3 hrs at RT. The absorbance of wells at 490 nm was measured using a microplate reader. Results were expressed as means (±sd) of quadruplicate wells obtained by subtraction from cell-free equivalents, to eliminate A_490_ produced by the media alone. Effects of pepper extracts on HCT116 cells were analysed by MTS assay expressed as a fold of control [%] of absorbance generated in cell-mediated MTS assays to the control group.

### Agilent × CELLigence real-time cell analysis

HCT116 cells (5 × 10^3^ cells/well) were seeded in 96-well plates (RTCA E-Plates 96) on *xCELLigence* RTCA systems (Agilent). The cells were treated with chilli extracts 24 hrs after seeding. HCT116cells were cultured in the absence or presence of tested drugs at concentrations ranging from 100 μM to 100 nM. The cell adhesion and spread without the manipulation of the cells were continuously monitored in 60 min intervals over the course of a 120 hrs observation period using the ×*CELLigence* RTCA system.

### Statistical analysis

Experiments under all conditions were performed in at least three independent measurements. Mean value and standard deviation were calculated using descriptive statistics. The data were analyzed by using the RTCA software Pro 1.2.1 (ACEA Bioscience). Statistical analysis was carried out by a non-parametric method, one-way ANOVA using SigmaPlot (Ver. 12.0). Differences were considered significant *p < 0.05; **p < 0.01; ***p < 0.001.

## Results and discussion

### Capsaicin and dihydrocapsaicin content in chili peppers

The exact content of CAP and DHK in presented different types of chili peppers has not yet been described in the literature; therefore, our results provide novel data. The findings showed the highest content of CAP and DHK in species TMS and BJ, while J and SP peppers had the lowest concentrations of CAP and DHK (Table 1). From the preparation of the experiment, the same conditions, including the same composition of soil and water, were maintained for the growth of pepper plants in the defined conditions. Every single aspect, such as differences in soil composition and chemical composition of water when cultivating plants in different ecological conditions of different areas of the world, could affect the content of individual components in plants. Environmental influences can also be considered the epigenetic factors that play a role in the expression of genes responsible for producing individual components, such as CAP, flavonoids, and phenolics. CAP, as a cancer preventive agent, shows wide applications against various types of cancer [50]. Studies have already determined its antiproliferative activity against HT-29 colon cancer cells and HepG2 liver cancer cells and high antioxidant activity and found high concentrations of CAP, flavonoids, phenolics, and total soluble solids content in nine different peppers belonging to *Capsicum annuum* (No. 1072 and cultivars CM334, A44750157, PM 217, Sunset, Grif 9285, PM 687) and *C. chinense* (CA4 and No.1745)*.* Line 1745 of *C. chinense* showed potential as a nutraceutical compound for the prevention and treatment of colon and liver cancers. The different levels of anticancerous phytochemicals, such as CAP and flavonoids, were also detected in cultivars [51]*.* The data indicate chili peppers with significantly higher CAP and DHK levels.

**Table 1:** Content of capsaicin and dihydrocapsaicin in chili peppers

Sample	Capsaicin [μM.ml^−1^] *concentration in the extract*	Dihydrocapsaicin [μM.ml^−1^] *concentration in the extract*
Habanero Red (HR)	1.667 ± 0.005	2.526 ± 0.013
Habanero Maya Red (HMR)	0.696 ± 0.005	0.613 ± 0.003
Trinidad Moruga Scorpion (TMS)	8.132 ± 0.023	9.843 ± 0.074
Jalapeno (J)	0.134 ± 0.001	0.333 ± 0.005
Serrano pepper (SP)	0.280 ± 0.003	0.744 ± 0.002
Habanero Red Savina (HRS)	1.187 ± 0.002	2.617 ± 0.005
Bhut Jolokia (BJ)	3.554 ± 0.002	2.755 ± 0.140
Jamaica Rosso (JR)	1.124 ± 0.005	1.944 ± 0.020

### Chili extracts and nuclease and topoisomerase activity

#### Nuclease activity

**Fig. 1:**
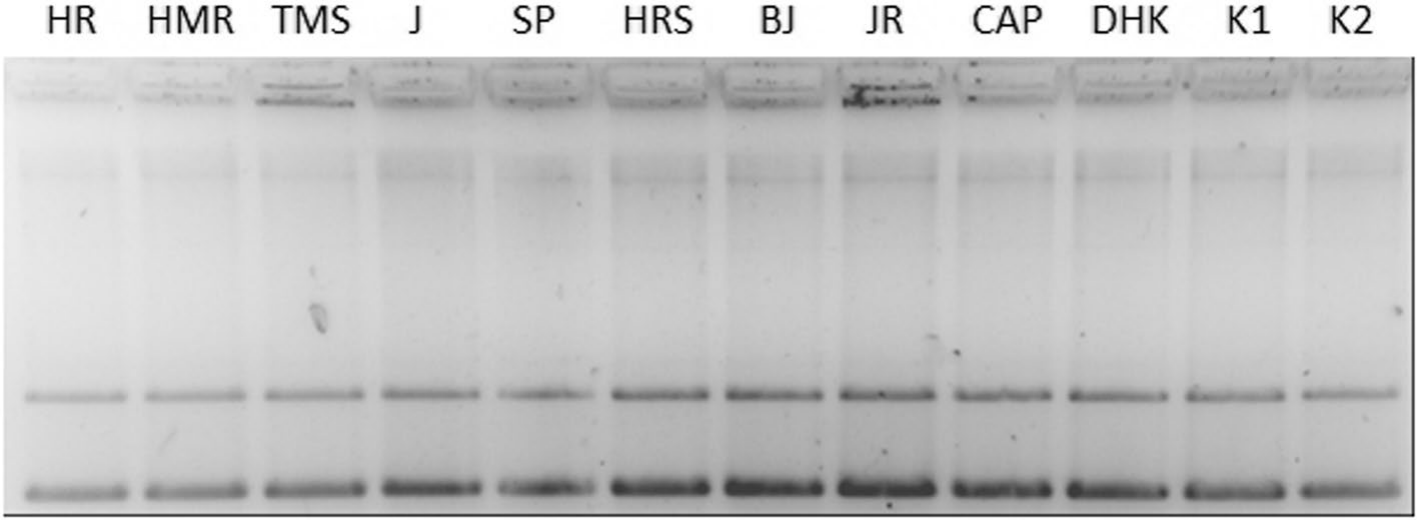
Nuclease activity. Nuclease activity of selected molecules were studied on isolated plasmid pUC19 and chili extracts in final concentration 1/10 of stock solution incubated at 37 °C for 18 hrs. Compounds of extract: HR – Habanero Red; HMR – Habanero Maya Red; TMS – Trinidad Moruga Scorpion; J – Jalapeno; SP – Serrano pepper; HRS – Habanero Red Savina; BJ – Bhut Jolokia; JR – Jamaica Rosso (final concentration 1/10 of stock solution); CAP – capsaicin (final concentration 0,05 mg/mL); DHK – dihydrocapsaicin (final concentration 0,05 mg/mL); K1 – pUC19; K2 – pUC19 + EtOH

A nuclease activity study confirmed that none of the samples could cleave plasmid DNA and that the ethanol content did not affect the plasmid (Fig. 1).

#### Decatenation assay for topoisomerase II

Human DNA Topo II is a nuclear enzyme that catalyzes the introduction of topological changes to the DNA molecule. hTopo II is effective in treating a wide spectrum of cancers. In the last decade, many scientists have designed, synthesized, and evaluated various bioactive molecules that target Topo II. In our experiment, we used a decatenation assay to measure Topo II’s catalytic activity to decatenate kinetoplast-catenated DNA (kDNA) in a cell-free system.

**Fig. 2:**
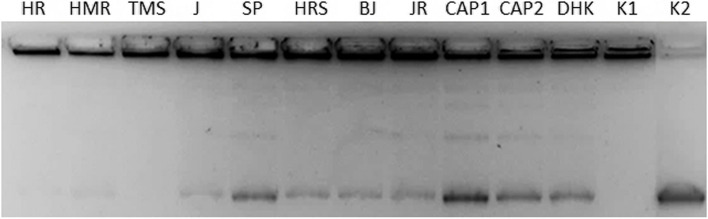
Decatenation assay for topoisomerase II. Topoisomerase II decatenation assay was carried out using kinetoplast DNA (kDNA, 200 ng) in human topoisomerase IIa (hTop IIa, 1.5 U) enzyme, in 1/10 of stock solutions (3 μL from extracts, capsaicin 0.5 mg/mL and 1.0 mg/mL). Extracts: HR – Habanero Red; HMR – Habanero Maya Red; TMS – Trinidad Moruga Scorpion; J – Jalapeno; SP – Serrano pepper; HRS – Habanero Red Savina; BJ – Bhut Jolokia; JR – Jamaica Rosso (final concentration 1/10 of stock solution); CAP 1 – capsaicin (final concentration 0.05 mg/mL); CAP 2 – capsaicin (final concentration 0.1 mg/mL); DHK – dihydrocapsaicin (final concentration 0.05 mg/mL); K1 – kDNA; K2 – kDNA + Topo II (1.5 U) + EtOH

**Fig. 3:**
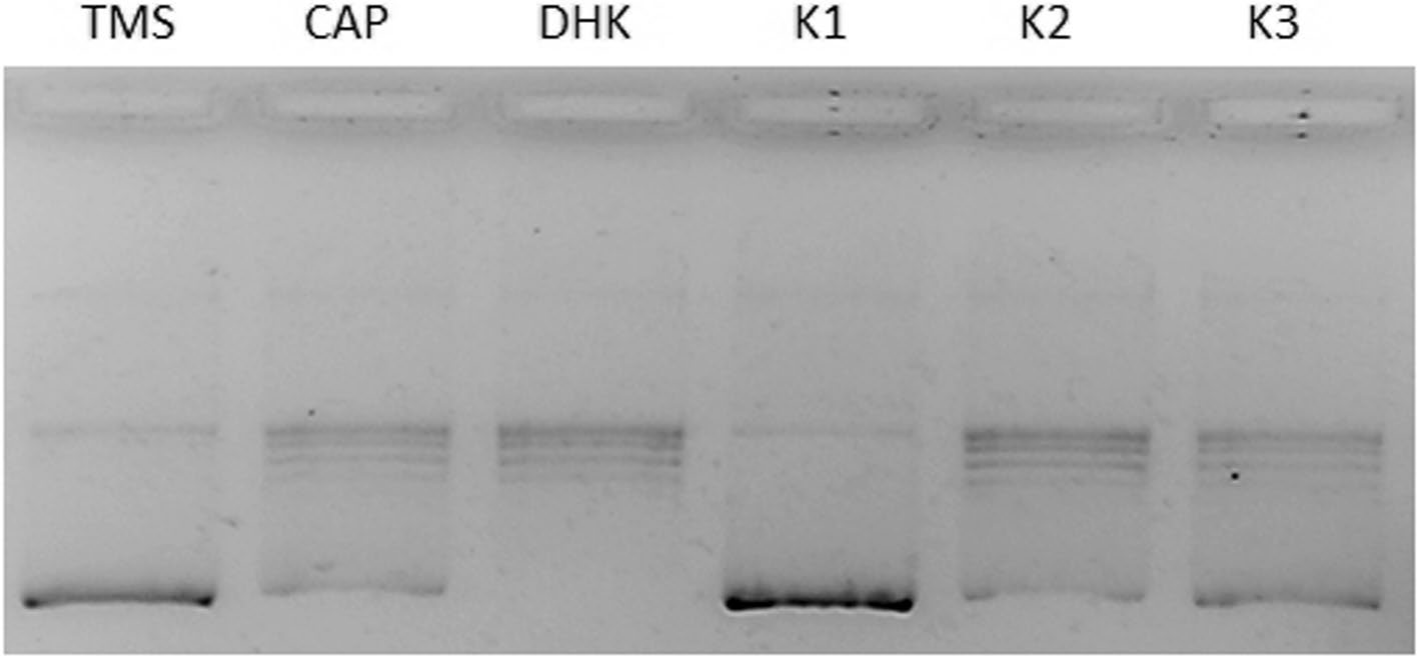
Relaxation assay for topoisomerase I. TMS – Trinidad Moruga Scorpion (final concentration 1/10 of stock solution); CAP – capsaicin (final concentration 0.05 mg/mL); DHK – dihydrocapsaicin (final concentration 0.05 mg/mL); K1 – pBR322; K2 – pBR322 + topo I (0.5 U); K3 – EtOH

**Fig. 4:**
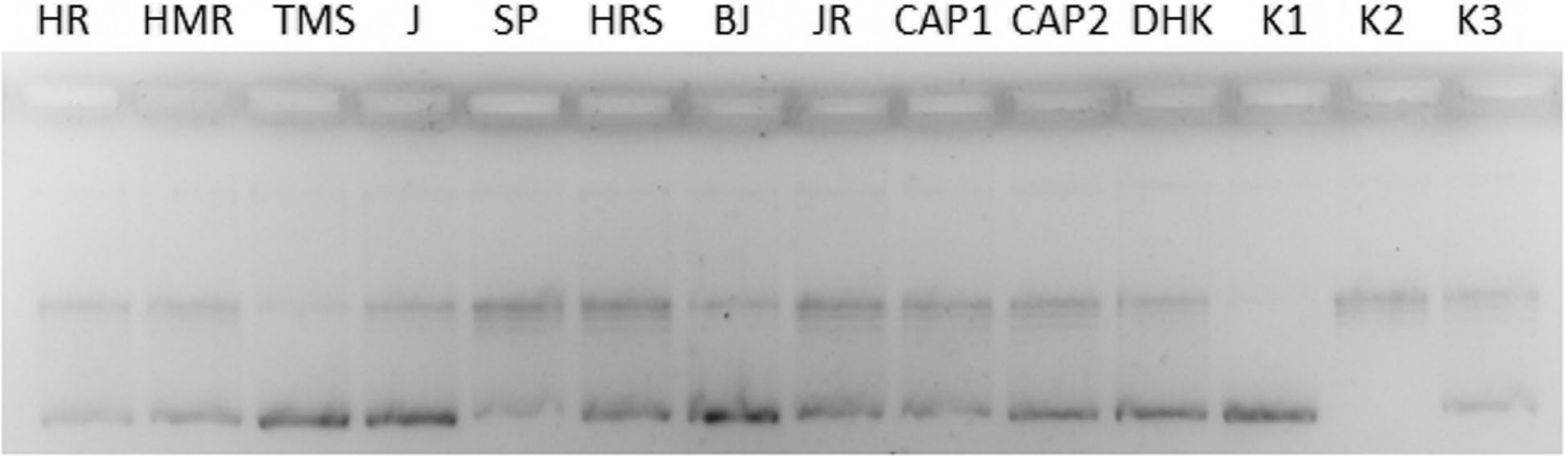
Relaxation assay for topoisomerase I. HR – Habanero Red; HMR – Habanero Maya Red; TMS – Trinidad Moruga Scorpion; J – Jalapeno; SP – Serrano pepper; HRS – Habanero Red Savina; BJ – Bhut Jolokia; JR – Jamaica Rosso (final concentration 1/10 of stock solution); CAP 1 – capsaicin (final concentration 1/10 of stock solution 0.05 mg/mL); CAP 2 – capsaicin (final concentration 0.1 mg/mL); DHK – dihydrocapsaicin (final concentration 0,05 mg/mL); K1 – pBR322; K2 – pBR322 + Topo I (1.0 U); K3 – pBR322 + Topo I + EtOH

At the beginning of the experiment, we first looked at whether ethanol (solvent) affects topo IIa and we found that it does not affect the activity of topo II topoisomerase (data not shown). However, a small non-significant effect of ethanol on the activity of topoisomerase IIa cannot be excluded, since the influence of ethanol on topoisomerase activity was demonstrated for topoisomerase I (Figs. 3 and  4). Figure 2 shows the inhibition of Topo II in all samples (Fig. 2), but complete inhibition was observed only for TMS, HR, and HMR. Samples J and SP had the lowest content of CAP and DHK substances, although they had approximately the same inhibition effect as in cases of CAP1 and CAP2 and DHK, suggesting that other substances present in the chili peppers might participate at inhibition activity of Topo II. Nevertheless, the presence of CAP and DHK is important in inhibiting the activity of this enzyme. As we observed a higher amount of catenated DNA in the wells of some samples, we assumed that ethanol could still affect the activity of Topo II.

#### Relaxation assay for topoisomerase I

The primary reaction of Topo I is the relaxation of supercoiled DNA which has a different electrophoretic mobility than a completely relaxed DNA. The effect of molecules on the relaxation ability of topoisomerase I was studied with human Topo I on plasmid pBR322. The TMS sample completely inhibited Topo I, while no activity was observed for CAP and DHK in the given conditions. For that reason, in the next step, we changed the hTopo I concentration from 0.5 U to 1.0 U (Figs. 3 and 4). We can observe that samples TMS, J, and BJ had the highest ability to inhibit Topo I (despite the weak effect of ethanol on Topo activity).

In contrast, SP, HR, and JR had no or little inhibitory activity, as they did not differ from K3 (ethanol probably played a role as a Topo inhibitor). The activity was also observed with CAP and DHK, with stronger inhibition of DHK. Based on the results, the TMS sample appeared to be the best candidate for further investigation, but it should also be noted that this sample contained much higher concentrations of CAP and DHK (approx. 800 μM and 900 μM) than other substances required for inhibition (as an example, acridine derivatives require less than 100 μM, ethidium bromide approximately 10 μM for Topo I, for acridines below 100 μM, and amsacrine below 250 μM for Topo II).

Based on the previous studies [49, 52, 53] class I (Topo poisons) and class II (catalytic inhibitors) DNA topoisomerase inhibitors were described, although not all showed toxicity to tumor cells. Many DNA Topo (class I) inhibitors are already commonly used in antitumor therapy: doxorubicin [54], cisplatin [55], and genistein [56]. The DNA Topo (class II) inhibitors, a plant alkaloid Camptothecin [57], its derivatives topotecan (Hycamtin) and irinotecan (CPT-11, Campostar), and another coumarin Topo inhibitor [58], are currently used in the clinic. Unfortunately, the clinical use of some of these anticancer drugs is limited due to their dose-limiting toxicity and chemical instability [48]. The non-camptothecin hTopo I inhibitors, indolocarbazoles (NB-506 and his derivative Edotecarin (J-107088), indenoisoquinolines (NSC 314622), indotecan (LMP400) and indimitecan (LMP776), and dibenzonaphthyridinones, have been investigated and under clinical development. These drugs have limitations due to their instability, severe side effects, and drug resistance caused by P-glycoprotein [59]. The catalytic activities of topoisomerases are modulated through their interactions with various proteins. The discovery of new anticancer drugs is important for many cancer patients resistant to specific drugs. DNA Topo remains an important therapeutic target of anticancer agents and antibacterial drugs [48]. We analyzed the nuclease activity, decatenation assay for Topo II, and relaxation assay for Topo I and found that the inhibition of DNA Topo I and IIa supported the use of pepper mix as a potential anticancer drug.

### Cytotoxic effect of chili extracts on HCT116 cells

**Fig. 5:**
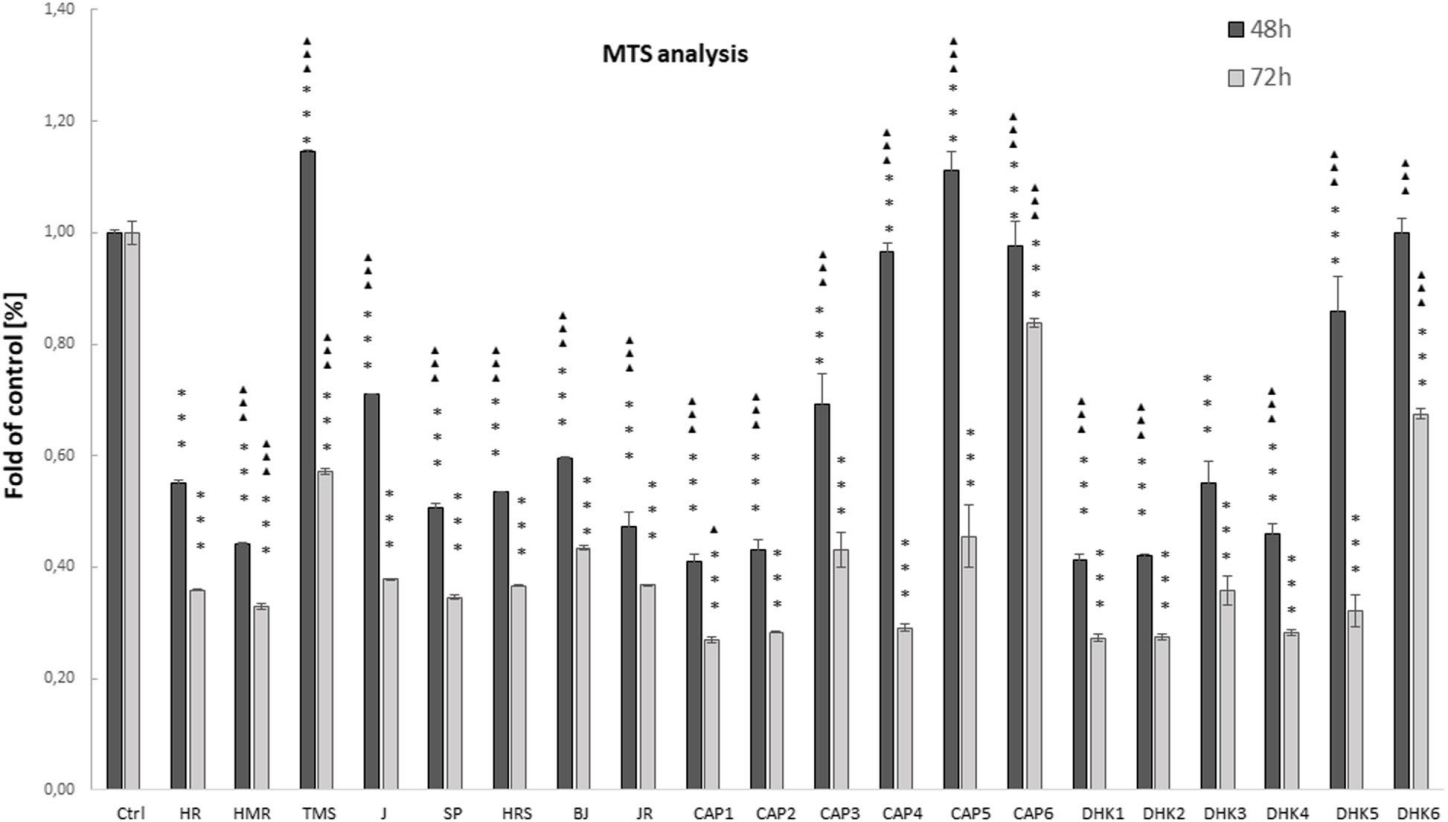
MTS Analysis. MTS analysis of cell viability at 48 and 72 hrs upon treatment with chili extracts (HR, HMR, TMS, J, S, HRS, BJ, JR) and capsaicin (CAP), dihydrocapsaicin (DHK) in different concentrations cultivated with HCT116 cells. The extracts of peppers HR, HMR, TMS, J, SP, HRS, BJ, JR. Control group (cells HCT116) was not affected by extracts, Cap1 - Cap6 concentrations of CAP at 10 μM, 25 μM, 50 μM, 100 μM, 150 μM, 200 μM. DHK1 - DHK6 concentrations of DHK at 10 μM, 25 μM, 50 μM, 100 μM, 150 μM, 200 μM. MTS analysis expressed as a fold of control [%] of absorbance generated in cell-mediated MTS assays to control group. Absorbance values are obtained from three independent experiments (*n* = 3 for each group) and values are expressed in mean ± SE. The groups treated with extracts alone were compared with control: * *p* < 0.05, **p < 0.01, ***p < 0.001. * vs Ctrl, ▲ vs HR and each extract mutually at both analyzed times (48 hrs, 72 hrs). Statistically insignificant [ns]: CAP1 vs DHK1, HR vs DHK3 at 48 hrs; CAP1 vs DHK1, BJ vs CAP3, HR vs DHK3, HRS vs JR at 72 hrs

The MTS method for the sensitive quantification of viable cells was used to assess the effect of chili extracts on HCT116 cell metabolic activity and/or viability. When comparing the cytotoxic effect of the pepper extracts with the effect of pure CAP and DHK on HCT116 cells, pure CAP and DHK had a comparable effect on cell viability (Fig. 5). TMS extract and CAP5 (150 μM) supported temporarily the viability of HCT116 cells in the observed time up to 48 hrs after administration of the substances to the cells. On the contrary, both TMS and CAP5 extracts reduced the viability of these cells at 72 hrs. In this regards, HR, HMR, J, S, HRS, BJ, JR extracts decreased tumor cell viability after treatment administration, demonstrated at 48 and 72 hrs in vitro*.*

The effects of extracts of TMS pepper (the content of CAP 122,30 ng/g) were comparable to that of CAP5. Other extract components likely contribute to the resulting biological effect on tumor cells. During the next 24 hrs of incubation, 72 hrs after adding the extracts to the cells, the effect of the extracts of all types of peppers, CAP (CAP 1 – 5), and DHK (DHK 1 – 5) significantly decreased the viability of HCT116 cells. Finally, the MTS assay demonstrated that chili extracts affect the cell viability of cancer cells at the concentrations used. Further analyses need to confirm these results by testing the antitumor effect of the individual components of the pepper extracts, mainly flavonoids and the contents of others substances.

### Antiproliferative effect of chili extracts on HCT116 cells

**Fig. 6:**
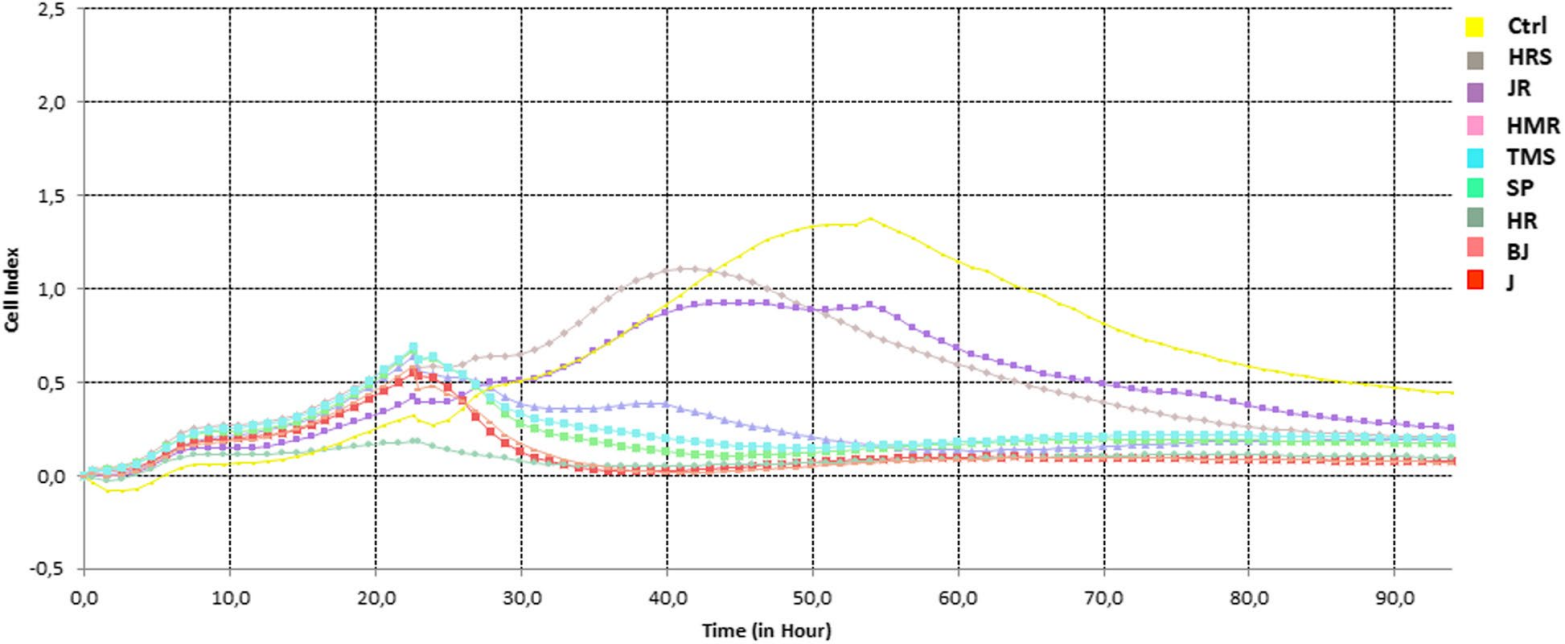
xCell proliferation. HCT116 cells (Ctrl) seeded in plates (RTCA E-Plates 96) were treated with 100 μM chilli extracts 24 hrs after seeding. The cell adhesion and spread were monitored over 90 hrs using the ×*CELLigence* RTCA system (Agilent). HRS and JR extracts (CAP content (362.57 and 343.40 μg/ml) increased cell proliferation during 30-50 hrs of culture

Cell culture assay (*xCELLigence* systems) showed the effect of all tested chili extracts on the suppression of the growth and proliferation of HCT116 cells at the end of the monitoring period (Fig. 6).

Despite the inconsistent effect of chili extracts in the first hours after their administration finally, the cell growth and/or proliferation decreased at a low level after treatment of each chili variety. HRS and JR extracts with a moderately high CAP content (362.57 and 343.40 μg/mL) increased cell proliferation during the first 30-50 hrs of culture, probably due to the influence of other components of the extract than the CAP content alone. Conversely, TMS and BJ extracts with higher CAP and DHK contents significantly reduced the proliferation of cancer cells several hours after the treatment. The results revealed TMS, J, and BJ pepper extracts have the highest ability to inhibit Topo I and cell proliferation, implying that they have the potential for medicinal treatment.

Recent studies have shown that pure CAP has antiproliferative and pro-apoptotic effects on different cancer cell lines and found an association of CAP at high doses with mutagenicity and carcinogenicity [60–62]. The research on pepper seed extract reported that it supresses the proliferation of human breast cancer cells MDA-MB-231 and MCF-7 cells [63]. CAP administration reduced cell proliferation and modulated the genes involved in cell proliferation, apoptosis, cell cycle suppression, and cancer tissue development and differentiation in male Wistar rats. CAP might have a chemopreventive effect against colorectal carcinogenesis [64]. Previously, the effect of chili extracts or pure CAP on colon cancers and HCT116 cells showed induced autophagy [65]. The CAP affects human colorectal cell lines LoVo and SW480 by inducing anti-tumorigenesis, deregulation of β-catenin/TCF-dependent signaling [45], cell death, and increased ROS and pro-apoptotic proteins in cells Colo205 [66]. Recently, the genome profile of Japanase chili pepper was sequenced and determined by Shirasawa et al. [67]. Stimulating food is one of the factor in the development of gastrointestinal tract cancers, with unclear association between chili pepper consumption and the risk of cancer. Chen et al. [68] found that geographic regions influence gastrointestinal cancer risk, particularly in Asian, African, and North American populations, which require greater attention during dietary counseling.

## Concluding remarks

Our pilot study pointed out for the first time the inhibitory effect of some chili extracts on Topo I and II activity, as well as the relationship of such extracts to the reduction of metabolic activity and proliferation of human colon cancer cell line HCT116. TMS pepper completely inhibited Topo I and thus is the best candidate for further investigation, suitable for medicinal treatments because their underlying mechanisms differ from those of Topo I inhibitors. In order to confirm the relationship between the inhibition of topoisomerase activity and the antitumor effect of chili extracts, it is necessary to test a wider range of tumor cells as well as the concentration and time scale of the action of such extracts.

### Supplementary Information

**Supplementary Material.**

## Data Availability

All data supporting the findings of this study are available within the paper and within its supplementary materials.

## Abbreviations

HR: Habanero red
HMR: Habanero maya red
TMS: Trinidad moruga scorpion
J: Jalapeno
SP: Serrano pepper
HRS: Habanero red savina
BJ: Bhut Jolokia
JR: Jamaica rosso
CAP: capsaicin
DHK: Dihydrocapsaicin
pBR: Common plasmid cloning vector in *E. coli* that is 4361 bp
Topo I: DNA topoisomerase I
Topo II: DNA topoisomerase II
kDNA: Kinetoplast DNA
MTS: Assay used to assess cell proliferation, cell viability and cytotoxicity
xCell assay: xCELLigence RTCA systems enable continuous, quantitative, real-time cell analysis
HCT116: Cell line isolated from the colon of an adult male with colon cancer
HPLC: High-performance liquid chromatography (HPLC), a technique in analytical chemistry
RPMI medium: a cell culture medium commonly used to culture mammalian cells
