# Supplementary material for: Chili pepper extracts, capsaicin, and dihydrocapsaicin as potential anticancer agents targeting topoisomerases
Source: BMC Complement Med Ther. 2024 Feb 21;24:96. doi: 10.1186/s12906-024-04394-5 (PMC10880293; doi:10.1186/s12906-024-04394-5)

**Table 2.**

**Results of the quantitative determination of dihydrocapsaicin in ethanol extracts from chili peppers processed by the program Chromeleono Dionex Version 7.2**


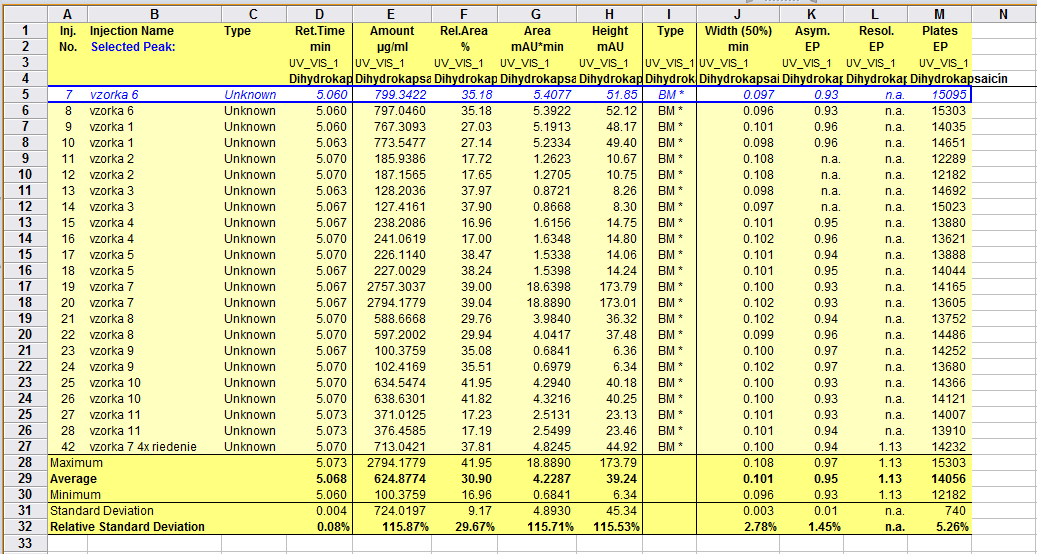

Supplement: Supplementary file 1 — Supplementary Material. [file 12906_2024_4394_MOESM1_ESM.zip › Suppl_Tab_2.docx]
