# Supplementary material for: Chili pepper extracts, capsaicin, and dihydrocapsaicin as potential anticancer agents targeting topoisomerases
Source: BMC Complement Med Ther. 2024 Feb 21;24:96. doi: 10.1186/s12906-024-04394-5 (PMC10880293; doi:10.1186/s12906-024-04394-5)
Supplement: Supplementary file 1 — Supplementary Material. [file 12906_2024_4394_MOESM1_ESM.zip › Supplementary_pictures_description.docx]

**Supplementary Table 1**

**Results of the quantitative determination of capsaicin in ethanol extracts from chili peppers processed by the program Chromeleono Dionex Version 7.2**

**Supplementary Table 2**

**Results of the quantitative determination of dihydrocapsaicin in ethanol extracts from chili peppers processed by the program Chromeleono Dionex Version 7.2**

**Supplementary Figure 1**

**Calibration curve for capsaicin (Chromeleono Dionex Version 7.2)**

**Supplementary Figure 2**

**Calibration curve for dihydrocapsaicin (Chromeleono Dionex Version 7.2)**

**Supplementary Figure 3**

**HPLC chromatogram of capsaicin and dihydrocapsaicin**
