# Supplementary figures and images for: Chili pepper extracts, capsaicin, and dihydrocapsaicin as potential anticancer agents targeting topoisomerases
Source: BMC Complement Med Ther. 2024 Feb 21;24:96. doi: 10.1186/s12906-024-04394-5 (PMC10880293; doi:10.1186/s12906-024-04394-5)

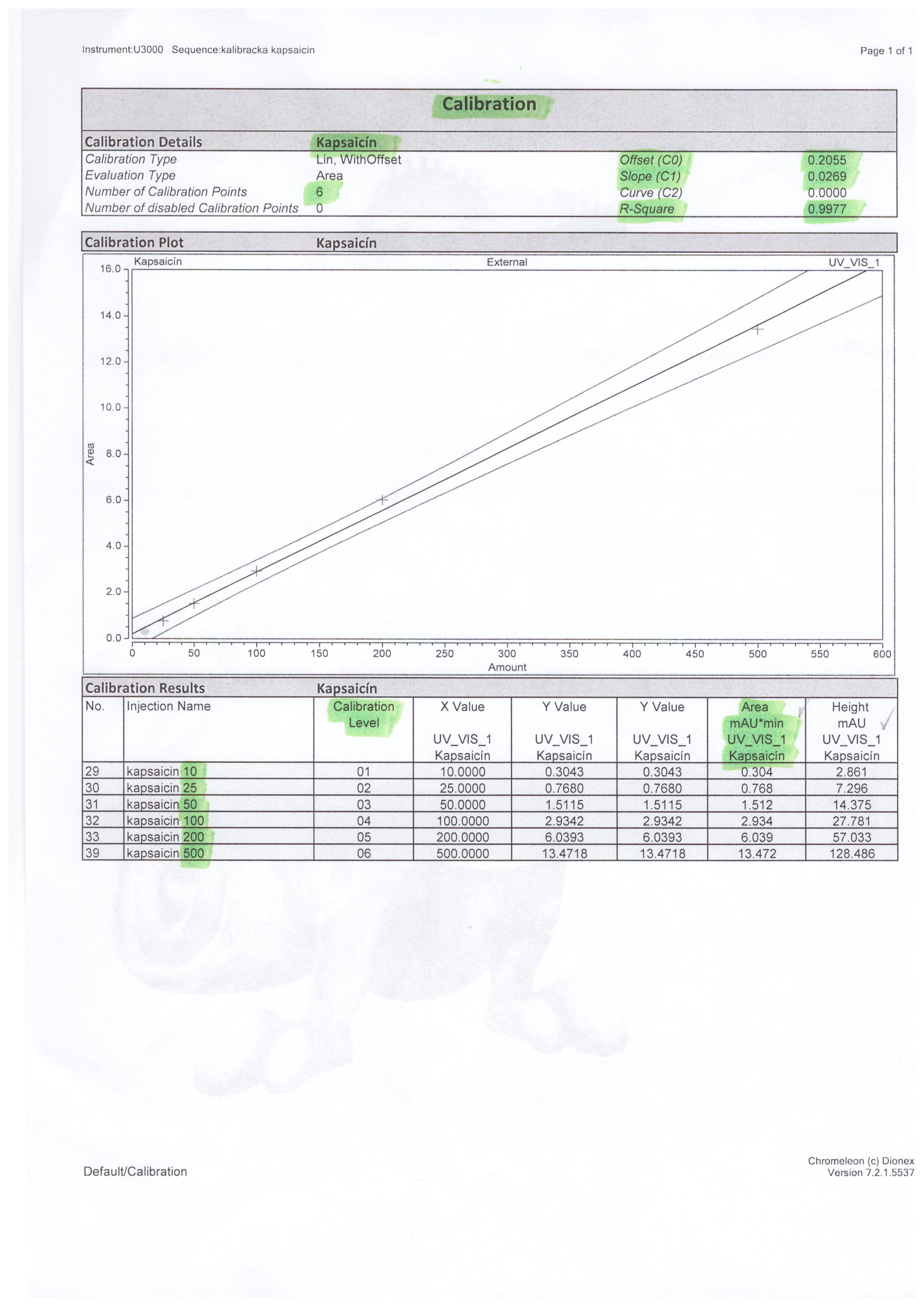

Supplement: Supplementary file 1 — Supplementary Material. [file 12906_2024_4394_MOESM1_ESM.zip › Suppl_Fig_1.jpg]

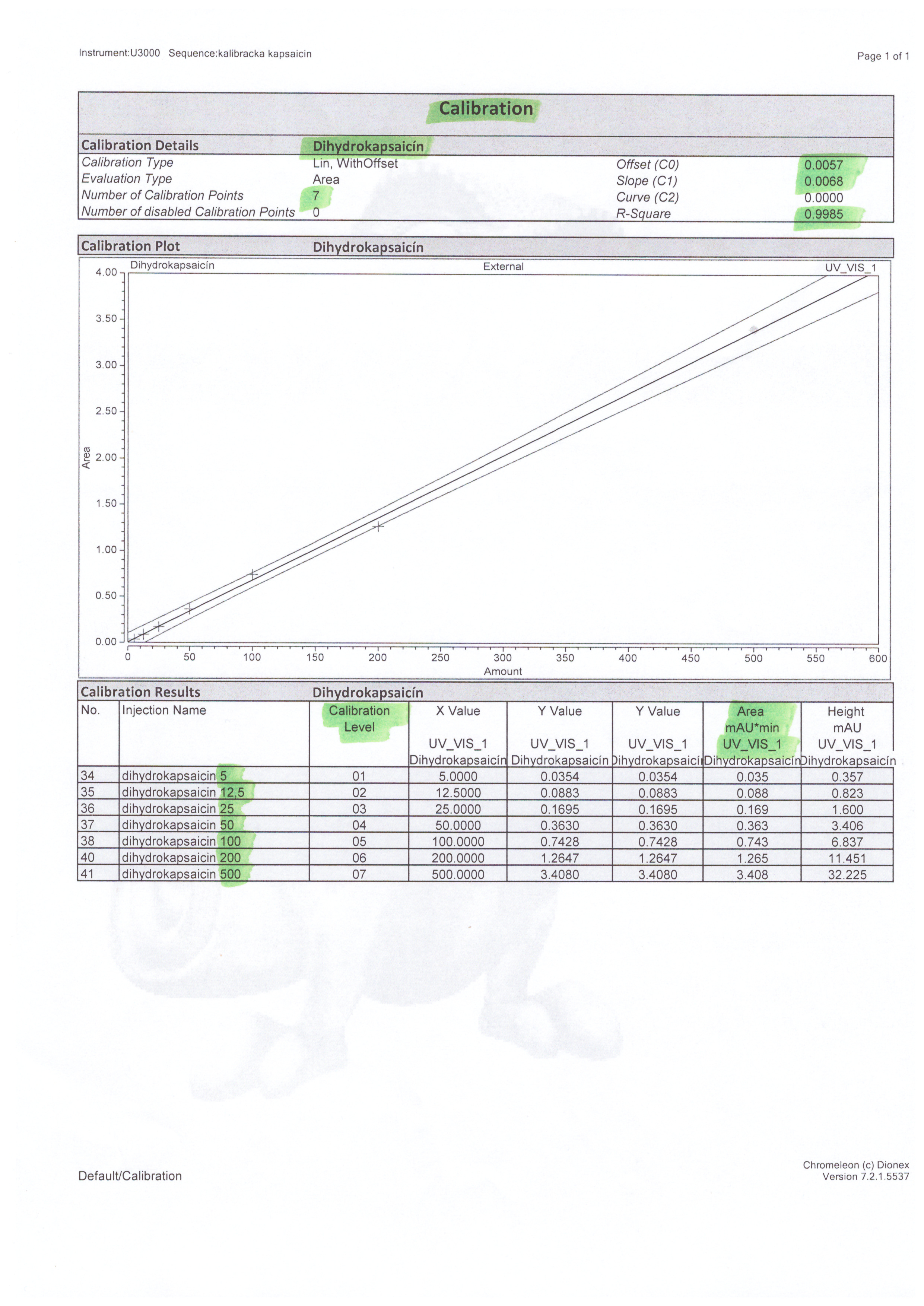

Supplement: Supplementary file 1 — Supplementary Material. [file 12906_2024_4394_MOESM1_ESM.zip › Suppl_Fig_2.jpg]

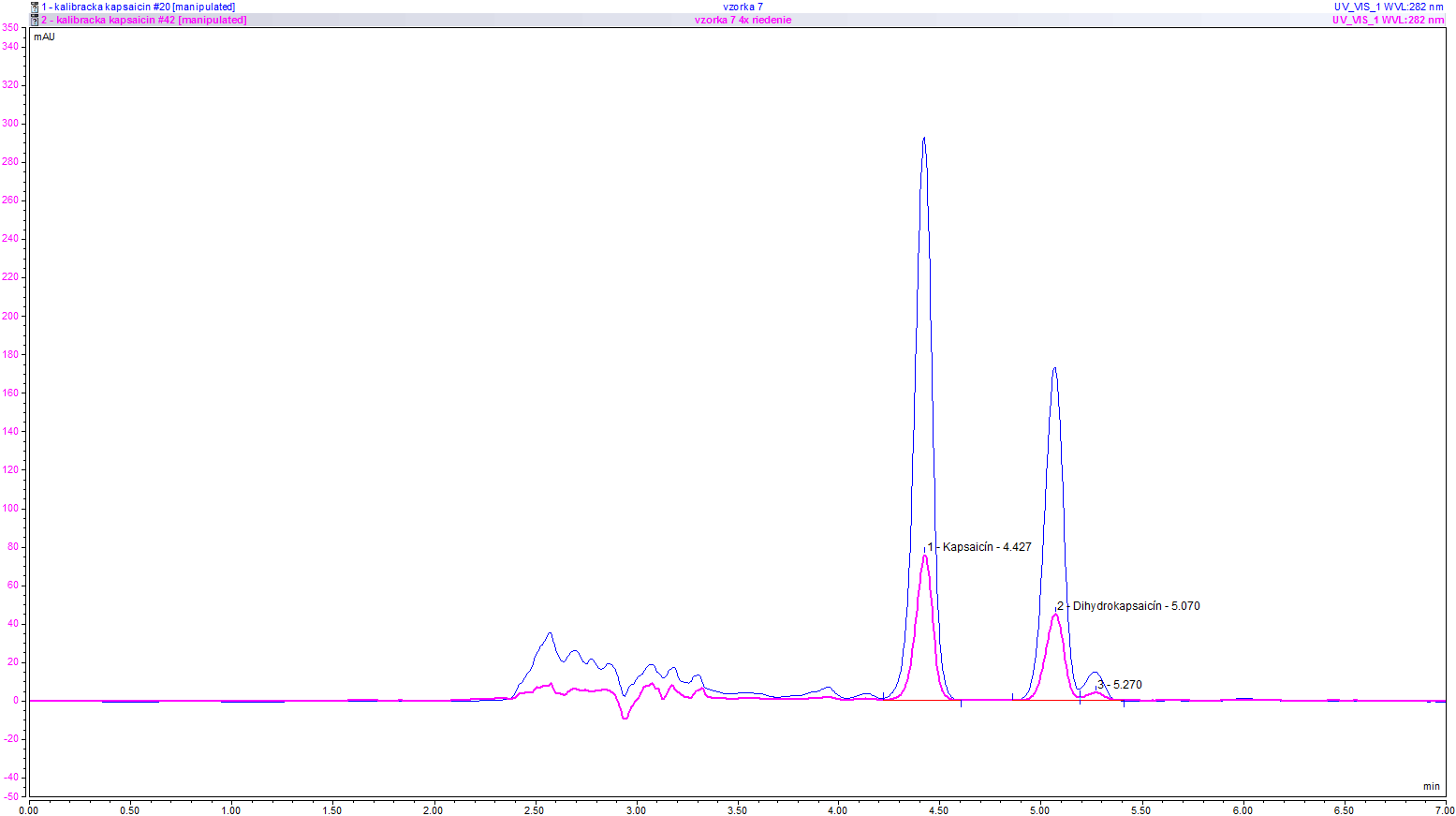

Supplement: Supplementary file 1 — Supplementary Material. [file 12906_2024_4394_MOESM1_ESM.zip › Suppl_Fig_3.png]
